# Genomic Characteristics of *Bifidobacterium thermacidophilum* Pig Isolates and Wild Boar Isolates Reveal the Unique Presence of a Putative Mobile Genetic Element with *tetW* for Pig Farm Isolates

**DOI:** 10.3389/fmicb.2017.01540

**Published:** 2017-08-15

**Authors:** Sayaka Tsuchida, Fumito Maruyama, Yoshitoshi Ogura, Atsushi Toyoda, Tetsuya Hayashi, Moriya Okuma, Kazunari Ushida

**Affiliations:** ^1^Laboratory of Animal Science, Graduate School of Life and Environmental Sciences, Kyoto Prefectural University Kyoto, Japan; ^2^Department of Microbiology, Graduate School of Medicine, Kyoto University Kyoto, Japan; ^3^Department of Bacteriology, Faculty of Medical Sciences, Kyushu University Fukuoka, Japan; ^4^Comparative Genomics Laboratory, National Institute of Genetics Mishima, Japan; ^5^Japan Collection of Microorganisms, RIKEN BioResource Center Tsukuba, Japan

**Keywords:** *Bifidobacterium thermacidophilum*, genome, pig isolate, *tetW*, recombinase

## Abstract

Genomic analysis was performed on seven strains of *Bifidobacterium thermacidophilum*, a *Sus*-associated *Bifidobacterium*. Three strains from the feces of domestic pigs (*Sus scrofa domesticus*) and four strains from the rectal feces of free-range Japanese wild boars (*S. s. scrofa*) were compared. The phylogenetic position of these isolates suggested by genomic analyses were not concordant with that suggested by 16S rRNA sequence. There was biased distribution of genes for virulence, phage, metabolism of aromatic compounds, iron acquisition, cell division, and DNA metabolism. In particular four wild boar isolates harbored fiber-degrading enzymes, such as endoglucanase, while two of the pig isolates obtained from those grown under an intensive feeding practice with routine use of antimicrobials, particularly tetracycline harbored a tetracycline resistance gene, which was further proved functional by disk diffusion test. The *tetW* gene is associated with a serine recombinase of an apparently non-bifidobacterial origin. The insertion site of the *tetW* cassette was precisely defined by analyzing the corresponding genomic regions in the other tetracycline-susceptible isolates. The cassette may have been transferred from some other bacteria in the pig gut.

## Introduction

Pig intestinal microbiota were investigated in terms of their functionalities in host health and growth promotion (Lee and Mazmanian, 2010; Brestoff and Artis, 2013). The development of pig intestinal microbiota, particularly the predominance of *Lactobacillus* spp., is considered a key event for health promotion (Konstantinov et al., 2006; Petri et al., 2010), and this lactobacillal predominance may relate to the significant reduction of Enterobacteriaceae and *Clostridium perfringens* (Inoue et al., 2005). Such development is affected by the weaning, dietary changes, and host-immune development (Katouli et al., 1997; Mackie et al., 1999; Inoue and Ushida, 2003; Ushida et al., 2008).

In addition to *Lactobacillus* spp., *Bifidobacterium* spp. are regarded as health-promoting bacteria in humans, because humans harbor *Bifidobacterium* as a predominant lactic acid bacteria (Mitsuoka and Kaneuchi, 1977; Matsuki et al., 2004). Especially, human breast-fed infants harbor high levels of *Bifidobacterium* spp., which may explain their low susceptibility to enteric diseases (Gibson and Wang, 1994; Roger et al., 2010; Guaraldi and Salvatori, 2012). Pigs do carry *Bifidobacterium* spp. as the primary component of intestinal microbiota, but to a lesser extent than *Lactobacillus* spp. as determined by both a culture-dependent method (Mikkelsen et al., 2003) and a culture-independent method (Hermann-Bank et al., 2013). We recently estimated the effect of domestication and modern pig feeding on the intestinal microbiota of Suidae by the metagenomic analysis of fecal DNA of various species of Suidae, such as *Sus scrofa scrofa, S. s. domesticus*, and *Potamochoerus porcus* (Ushida et al., 2016). In that study, we showed that *Bifidobacterium* was a predominant member (top 10 primary member) in the gut microbiota of wild boars and, to a lesser extent (top 20 member), in domestic pigs. Since *Lactobacillus* spp. were at the top 20 level of bacterial genera in wild boars, domestication and a modern feeding system may have induced the predominance of *Lactobacillus* spp. over *Bifidobacterium* spp. in pigs. Although the reasons for such a substitution are unrevealed, genomic analyses of Suidae-associated *Bifidobacterium* may detect the genetic changes associated with the adaptation to domesticated hosts and/or those grown in an industrialized pig-production system. For such study, we need to identify and isolate the Suidae-associated *Bifidobacterium* carried by both wild boars and domestic pigs.

Many strains of *Lactobacillus* spp. and *Bifidobacterium* spp. have been sequenced so far (Kant et al., 2011; Drissi et al., 2014; Lugli et al., 2014). But *B. thermacidophilum* intraspecies comparison has not been performed yet.

In this study, we analyzed the genomes of seven strains of *B. thermacidophilum*, which were isolated from pigs and wild boars. We found several fundamental differences in their draft genome sequences between the isolates from domestic pigs and those from wild boars. Although the number of strains tested here was limited, the difference in the draft genome sequences suggested the adaptation of Suidae-associated *Bifidobacterium* to the conditions in wild and artificial feeding.

Antimicrobial resistance (AMR) is now well-recognized as the primary threat to public health (O'Neill, 2014), and animal agriculture is considered an important industrial sector in which AMR continuously emerges due to antimicrobial use that promotes clinical and prophylactic growth (Woolhouse and Ward, 2013). Accordingly, the propagation of AMR of animal origin through the food chain has been routinely discussed. Probiotics have been regarded as a potential alternative for prophylactic drugs in animal agriculture (de Vrese and Schrezenmeir, 2008). However, since human-associated and probiotic strains of *Bifidobacterium* spp. are resistant to a range of antibiotics, including tetracycline (Moubareck et al., 2005), the transmission of AMR from probiotic strains to other bacterial members of intestinal microbiota could occur (Van Hoek et al., 2008). In this study, in addition to the biased distribution of genes responsible for carbohydrate and nitrogen metabolism in seven *B. themacidophilum* strains, resistance to antimicrobials was investigated.

## Materials and methods

### Strain isolation and identification

Bifidobacteria were isolated from fresh feces of animals using *Bifidobacterium*-selective (BS) agar plates under anaerobiosis as previously described (Tsuchida et al., 2014). Species identification was made based on the results of their 16S rRNA gene sequences as previously described (Tsuchida et al., 2014).

Strains 45 and 53 were isolated from the freshly defecated feces of a growing piglet and a sow, respectively, in a commercial pig farm in Aichi Prefecture, Japan. In that commercial farm, piglets were fed on commercial formula feed supplemented with avilamycin, colistin sulfate, and morantel citrate for growth promotion. In addition, chlortetracycline was routinely dosed (at 400 ppm in feed) to the growing piglets to prevent respiratory infections such as mycoplasmosis. All sows in this commercial farm also received veterinary interventions of antibiotic dosing when respiratory and enteric disease symptoms were observed. Strain 194 was isolated from the freshly defecated feces of a sow kept in the experimental farm of Kyoto Prefectural University, Kyoto, Japan. Strains 195, 196, 197, and 198 were from the rectal feces of wild boars hunted in a mountain area in Japan. Strain 195 was from a wild boar in Gunma Prefecture in November 2011, and the remaining three were from wild boars in Shiga Prefecture between December 2012 and March 2013. These strains were previously identified by partial 16S RNA gene sequencing according to the method described in Tsuchida et al. (2014). Phylogenetic tree was finally constructed with full length sequences of 16S rRNA gene, which were assembled after whole genome sequencing (Takemura et al., 2017). In addition, the average nucleotide identities (ANIs) were estimated between these seven isolates, together with reference strains [*B. thermophilum* JCM 1207^T^, *B. thermacidophilum* subsp. *thermacidophilum* LMG 21395^T^ (= JCM 11165^T^), *B. thermacidophilum* subsp. *porcinum* LMG 21689^T^ (= JCM 16945^T^), and *B. thermophilum* RBL67], according to Goris et al. (2007).

### Genome sequencing and *de novo* assembly

Genomic DNA was extracted from the seven strains using QuickGene Mini 80 (Kurabo, Tokyo, Japan) after 24-h culture on Eggerth–Gagnon (EG) agar (BBL, Cockeysville, MD, USA) plates with a supplementation (5%) of horse defibrinated blood (Nihon Biotest, Tokyo, Japan) at 37°C. Colonies were collected into the lysis buffer (MDT buffer) of QuickGene Mini 80. Extracted DNA was subjected to library construction (average insert sizes: 400–500 bp) using a Nextera DNA Library Preparation Kit (Illumina, San Diego, CA, USA), and multiplex paired-end sequencing was performed with the HiSeq platform with a TruSeq SBS Kit v3-HS v.2.0 (2 x 151 cycles) (Illumina). Base call was achieved by RTA1.17.21.3 (Illumina). Generated read sequences were assembled after trimming a low-quality sequence using Trimmomatic (Bolger et al., 2014) by Velvet (Zerbino, 2010) after the optimization of k-mer (Table 1). Genomic sequences obtained in this study were submitted to the DNA data bank of Japan (DDBJ) with accession numbers DRX061137, DRX061141, DRX061144, DRX061148, DRX061152, DRX061156, and DRX061160.

**Table 1:** Genome structure of strains of *Bifidobacterium thermoacidophilum*.

**Source**	**Strain**	**Genome size**	**GC%**	**CDS**	**N50 (bp)**	**Scaffolds number**	**Best k-mer**	**Coverage**	**Accession no**.
Pig_Aichi	45	2,076,303	60.1	1,686	225,166	35	97	379	DRX061137
Pig_Aichi	53	2,073,900	60.1	1,696	229,061	30	81	254	DRX061141
Pig_KPU	194	2,534,183	60.5	2,104	77,635	109	85	278	DRX061144
Wild boar Gunma	195	2,452,931	60.3	1,949	126,996	59	97	333	DRX061148
Wild boar Shiga	196	2,355,141	60.4	1,853	146,723	59	79	341	DRX061152
Wild boar Shiga	197	2,356,693	60.4	1,843	157,868	72	71	328	DRX061156
Wild boar Shiga	198	2,353,043	60.5	1,854	137,407	64	75	410	DRX061160

*Reference strain: Bifidobacterium thermophilum RBL67. CDS: protein coding sequence*.

### Gene annotation and construction of a phylogenetic tree

The annotations of the reference genomes and the seven genomes sequenced in this study were automatically processed using the Rapid Annotations using Subsystems Technology (RAST) server (Aziz et al., 2008). Pan-genomic analysis was done according to Maruyama and Ueki (2016). In brief, pan-genomic analysis was conducted using PGAP software using cutoff values of 50% identity and an E-value < 10^−5^ (Zhao et al., 2012). In this analysis, orthologs in each strain in the dataset were determined by all-vs.-all BLASTP search followed by the Markov Cluster Algorithm (MCL), and phyletic inference was calculated by the neighbor-joining method based on the presence/absence matrix of the orthologs in each combination of the strains (Zhao et al., 2012). All CDSs were, accordingly, clustered into homology groups by using the gene-family method implemented in the pan-genome analysis pipeline (PGAP-1.01) (Zhao et al., 2012) with default parameters and employing the MCL algorithm (Enright et al., 2002).

Phylogenetic analysis was done according to Minegishi et al. (2015). In brief, the amino acid sequence of the single-copy core CDSs was concatenated and used for the construction of a maximum-likelihood phylogenetic tree with 100 bootstrap iterations. The sequences showing any signs of recombination in a Phi test (cutoff value: *P* ≥ 0.05) were excluded. In addition, we used kSNP program version 3.021 (Gardner et al., 2015), an alignment-free sequence analysis tool, to build whole-genome phylogenies based on single nucleotide polymorphisms (SNPs) in the whole-genome data of all seven strains and reference strains. For SNP determination, we applied default parameters and a k-mer selected as the optimal value predicted by the kSNP-associated Kchooser script (Gardner et al., 2015). A maximum-parsimony tree based on all of the SNPs was constructed. The pan-genome dendrogram was also constructed based on the presence/absence of all the homology groups assigned by the PGAP pipeline in this study. The tree was visualized by a Dendroscope 3 (Huson and Scornavacca, 2012).

### Prediction of antibiotic resistance genes and insertion sequences

Antibiotic resistance genes were predicted in the protein sequence data of presently assembled draft genomes and those in the WGS sequence data of reference strains (*B. thermophilum* JCM 1207^T^, *B. thermacidophilum* subsp. *thermacidophilum* JCM 11165^T^, and *B. thermacidophilum* subsp. *porcinum* JCM 16945^T^) by the Resistance Gene Identifier (RGI) in the Comprehensive Antibiotic Resistance Database (CARD) (McArthur et al., 2013). Insertion sequences (ISs) were identified by an IS finder (Siguier et al., 2006). In addition, genomic sequences around the *tetW* gene were compared using a GenomeMatcher to reveal the insertion of a putative *tetW* mobile cassette (Ohtsubo et al., 2008).

### Disk diffusion test

In addition to the seven strains (45, 53, 194, 195, 196, 197, and 198), three type strains, *B. thermophilum* JCM 1207^T^, *B. thermacidophilum* subsp. *thermacidophilum* JCM 11165^T^, and *B. thermacidophilum* subsp. *porcinum* JCM 16945^T^, obtained from the RIKEN BioResource Center, were tested for their susceptibility to tetracycline, nalidixic acid, ofloxacin, fosfomycin, rifampicin, erythromycin, and lincomycin. Morantel citrate and colistin sulfate were tested by disk diffusion because avilamycin, morantel citrate, and colistin sulfate were mixed with feed, and suckling piglets were exposed to these feed additives. Since the distribution of avilamycin was limited to users approved by the Food and Agricultural Materials Inspection Center, we did not determine susceptibility in this study.

The cells were grown on a blood and liver (BL) agar (Nissui Pharmaceutical Co. Ltd., Tokyo, Japan) plate supplemented with 5% horse defibrinated blood under anaerobiosis at 37°C. A portion of the colony was taken and suspended in Mueller Hinton broth (Becton, Dickinson and Company, Sparks, MD, USA) to a McFarland 1.0 turbidity standard. Since the method and criteria to evaluate the antibiotic resistance of the genus *Bifidobacterium* have not been standardized, we performed antibiotic susceptibility tests using BL agar plates supplemented with 5% (v/v) horse defibrinated blood in place of Mueller Hinton agar plates, with a higher cell density of inocula (adjusted to a 1.0 McFarland turbidity standard). Tetracycline, nalidixic acid, ofloxacin, erythromycin, fosfomycin, rifampicin, lincomycin, and colistin sulfate disks were obtained from Eiken Chemical Co. Ltd., (Tochigi, Japan) and used according to the manufacturer's instructions. The inhibition zone was determined after 18 h of culture. Since a morantel citrate disk was not commercially available, a 4-mm disk containing 10 μg (titer) of morantel (Wako Pure Chemicals, Osaka, Japan) was applied to the disk diffusion test as above.

### Ethics

The experiment was approved by the Kyoto Prefectural University Experimental Animal Committee (KPU240410). Sampling of pig feces was achieved in a non-invasive manner. Accordingly, access to the feces was allowed under direction of the farms, with verbal permission. In the case of wild boars, we followed hunting groups to collect rectal samples from the hunted wild boars. All of the hunters obtained permission from the national and local governments to hunt wild boars.

## Results

### Identification of *Bifidobacterium* isolates

**Figure 1:**
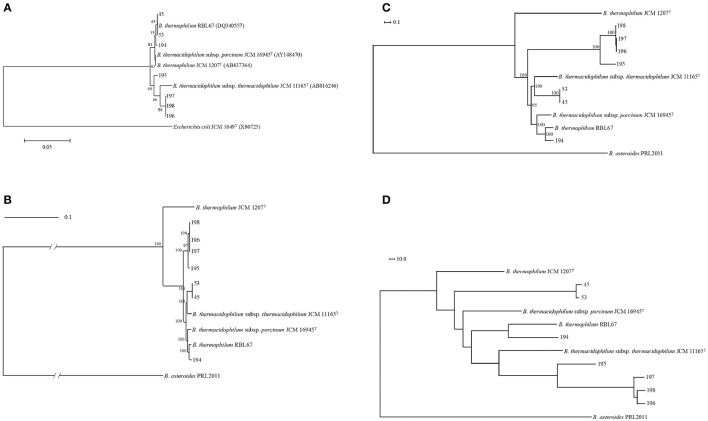
Phylogenetic relationship of *Bifidobacterium thermacidophilum* strains of various origins. **(A)** Neighbor-joining phylogenetic tree of 16S rRNA of *Bifidobacterium thermacidophilum* strains of various origins with reference strains, **(B)** A maximum likelihood phylogenetic tree of core CDSs of *Bifidobacterium thermacidophilum* strains of various origins with reference strains, **(C)** A consensus parsimony tree based on all of the SNPs of *Bifidobacterium thermacidophilum* strains of various origins with reference strains, **(D)** A neighbor-joining dendrogram of the presence/absence matrix of the orthologs of *Bifidobacterium thermacidophilum* strains of various origins with reference strains. The figures denote the strain numbers.

In this study, seven bifidobacterial strains were isolated from pigs or wild boars. According to 16S rRNA phylogeny, isolates were separated into two clusters: one was composed of pig isolates (isolates 45, 53, and 194) identified as *B. thermacidophilum* subsp. *porcinum* with a 99% identity of the 16S rRNA sequence, and the other was composed of wild boar isolates (isolates 195, 196, 197, and 198) identified as *B. thermacidophilum* subsp. *thermacidophilum* with a 98–99% identity of the 16S rRNA gene sequence. According to the 16S rRNA phylogenetic tree, the former group was also close to *B. thermophilum* RBL67 (Figure 1A), and this latter character strain, RBL 67, was unexpectedly separated from its type strain *B. thermophilum* JCM 1207^T^.

### Genome structures and genome-based phylogenetic analyses

The features of genomes and sequencing statuses of the presently analyzed seven strains are shown in Table 1. The genome sizes, G+C contents, and numbers of CDSs of these isolates range from 2.1 to 2.5 Mb, from 60.1 to 60.5 (%), and from 1,686 to 2,104, respectively (Table 1). ANI values are shown in Table S1. The ANIs between strain 45, 53, and 194 were in the range of 95–100%. These three strains all showed ANI values larger than 95% with JCM 16945, JCM 11165, and RBL67. Wild boar isolates, 195, 196, 197, and 198, showed an ANI value larger than 95% in each combination. These wild boar isolates showed an ANI value smaller than 95% with all laboratory strains.

Pan-genome analyses resulted in 773 core gene clusters (common for all strains analyzed) and 4,690 dispensable gene clusters. A maximum likelihood phylogenetic tree of concatenated single-copy core CDSs shows the three clusters: 45 and 53 with *B. thermacidophilum* subsp. *thermacidophilum* JCM 11165; 194 and RBL 67 with *B. thermacidophilum* subsp. *porcinum* JCM 16945; and strains 195, 196, 197, and 198 (Figure 1B). RBL 67 was unexpectedly separated from the type strain of *B. thermophilum* JCM 1207^T^. A similar tendency was observed in the SNP-based consensus parsimony tree in which three clusters (pig isolate 194 and RBL 67 with JCM 16945; pig isolates 45 and 53 with JCM 11165; and boar isolates 195, 196, 197, and 198) are shown (Figure 1C). Wild boar isolates 195, 196, 197, and 198 were in the independent clade, although they were identified as *B. thermacidophilum* subsp. *thermacidophilum* according to 16S rRNA sequence similarity (Figure 1A). These phylogenetic trees based on the draft genome sequences dis not completely match that of 16S rRNA sequences.

A dendrogram constructed using pan-genomic gene repertoires (Figure 1D) also showed the separation between pig isolates 45, 53, and 194 and wild boar isolates 195, 196, 197, and 198. The separation of pig isolates, *B. thermacidophilum* subsp. *porcinum*, from wild boar isolates, *B. thermacidophilum* subsp. *thermacidophilum*, depends on the biased distribution of genes with regard to the virulence, phage, metabolism of aromatic compounds, iron acquisition, cell division, and DNA metabolism (Table S2). Gene clusters distributed with a strong bias for either pig isolates or wild boar isolates are listed in Table S3, in which 65 gene clusters were distributed to pig isolates (45, 53, and 194) more than wild boar isolates (195, 196, 197, and 198) and 122 gene clusters were distributed to wild boar isolates more than pig isolates. However, within the present three pig isolates, all three strains shared only 27 gene clusters among 65 gene clusters; 38 gene clusters were shared only by strains 45 and 53. In addition, their type strain, JCM 16945, did not harbor 50 gene clusters of the 65 genes clusters. In fact, among 38 gene clusters shared only in strains 45 and 53, seven gene clusters were detected in type strain JCM 11165.

For the gene clusters distributed mostly to wild boar isolates, 81 gene clusters were evenly shared by all four strains, but their type strain JCM 11645 shared 56 gene clusters. There were several distinct gene clusters in that biased distribution. Putative mobile element genes were suggested to be present in all of the strains analyzed, but distributed more in pig isolates and the pig origin laboratory strain (JCM 16945). Proteins related to the Type I restriction system also showed such biased distribution in the present pig isolates. As for carbohydrate metabolism, starch-related enzymes such as maltodextrin glucosidase and alpha-glucosidase were detected in pig isolates together with pig-derived laboratory strains. In contrast to these pig isolates, genes of endoglucanase, beta-glucosidase, chitinase, an endoxylanase-related protein, beta-mannosidase, and pullulanase were detected in strains 195, 196, 197, and 198, as well as in JCM 16945 with the exception of pullulanase. This latter enzyme was not detected in JCM 16945. Ferric ion transporters were detected in four wild boar isolates. These gene clusters were not detected in pig isolates except for JCM1201 in some cases.

Pilin-related proteins were detected in the above-mentioned wild boar isolates and JCM 16945, but one pig isolate, 194, also harbored these gene clusters except for the twitching motility protein PilT. Gene clusters for the phage-related protein were only detected in the present four wild boar isolates. Other various gene clusters detected in four wild boar isolates were not shared in the laboratory strains in most cases.

### Drug resistance genes and confirmation by disk diffusion test

A gene cluster for an ABC-type multidrug transport system, which may relate to the drug resistance, was detected in 194, 195, 196, 197, and 198 in addition to all of the laboratory strains. Genes for virulence and defense, such as the lincomycin-resistant protein, tetracycline resistance gene, *tetW*, and mercuric ion reductase, were found only in strains 45 and 53. In addition to SEED, a range of resistance genes was suggested by the CARD (Table 2). According to the CARD, bifidobacterial intrinsic *ileS* and EF-Tu mutation, *alaS* and *rpoB* were suggested to be harbored in all isolated strains. Genes conferring resistance to fluoroquinolone, tetracycline, macrolide, fosfomycin, and colistin were suggested for certain isolates.

**Table 2:** CARD and SEED suggestion about the presence of putative resistance gene and the results of disk diffusion test.

**Gene/Strain no**.	**45**	**53**	**194**	**195**	**196**	**197**	**198**	**JCM 1207**	**JCM11165**	**JCM16945**
Bifidobacteria intrinsic *ileS* conferring resistance to mupirocin	+	+	+	+	+	+	+	+	+	+
*Escherichia coli* EF-Tu mutants conferring resistance to kirromycin	+	+	+	+	+	+	+	+	+	+
*Mycobacterium tuberculosis gy*rA conferring resistance to fluoroquinolones	+	+	+	+	–	–	+	+	+	+
*Mycobacterium tuberculosis gyr*B mutant conferring resistance to fluoroquinolone	+	+	–	–	–	+	–	–	–	–
Aminocoumarin resistant *alaS*	+	+	+	+	+	+	+	+	+	+
*mfd* antibiotic target protection protein; fluoroquinolone	+	+	+	+	+	+	+	+	+	+
*Staphylococcus aureus rpoB* mutants conferring resistance to rifampicin	+	+	+	+	+	+	+	+	+	+
*Chlamydia trachomatis* murA	+	+	+	–	+	+	+	+	+	+
*tetT (tetS, tetT, tetW, tet32, tetM, tetO, tet36, tet44)*	+	+	–	–	–	–	–	+	–	–
*PmrE*	–	–	–	+	+	+	+	–	–	–
*desR*	–	–	–	+	+	+	+	–	–	+
**DISK DIFFUSION TEST**										
Tetracycline	R	R	S	S	S	S	S	R	S	S
Nalidixic acid	R	R	R	R	R	R	R	R/S	R	R
Ofloxacin	S	S	S	S	S	S	S	S	S	S
Fosfomycin	R	R	S	S	S	S	S	S	S	S
Rifampicin	S	S	S	S	S	R	R	R	S	S
Erythromycin	S	S	S	S	S	S	S	S	S	S
Colistin sulfate	R	R	S	S	S	R	R	S	S	S
Lincomycin	S	S	S	S	S	S	S	S	S	S
Morantel citrate	S	S	S	S	S	R	R	R	S	S

*The putative resistance genes are suggested by CARD and SEED. Disk diffusion test was conducted by tetracycline, nalidixic acid, ofloxacin, fosfomycin, rifampicin, erythromaycin, colistin, and lincomycin disk (Eiken Chemical). Morantel citrate disk was prepared by authors. Details, see text*.

The disk diffusion test confirmed the resistance of pig isolates 45, 53, and JCM11165 to tetracycline. However, other resistances suggested by the CARD or SEED were not concordant with disk diffusion test. In fact, all strains were resistant to nalidixic acid, but they all were sensitive to ofloxacin. None of the strains tested showed resistance to lincomycin or erythromycin according to the disk diffusion test. Resistance to fosfomycin was confirmed only in strains 45 and 53. Furthermore, resistance to rifampicin was confirmed in strains 197, 198, and JCM 1207.

### Serine recombinase and *tetW*

**Figure 2:**
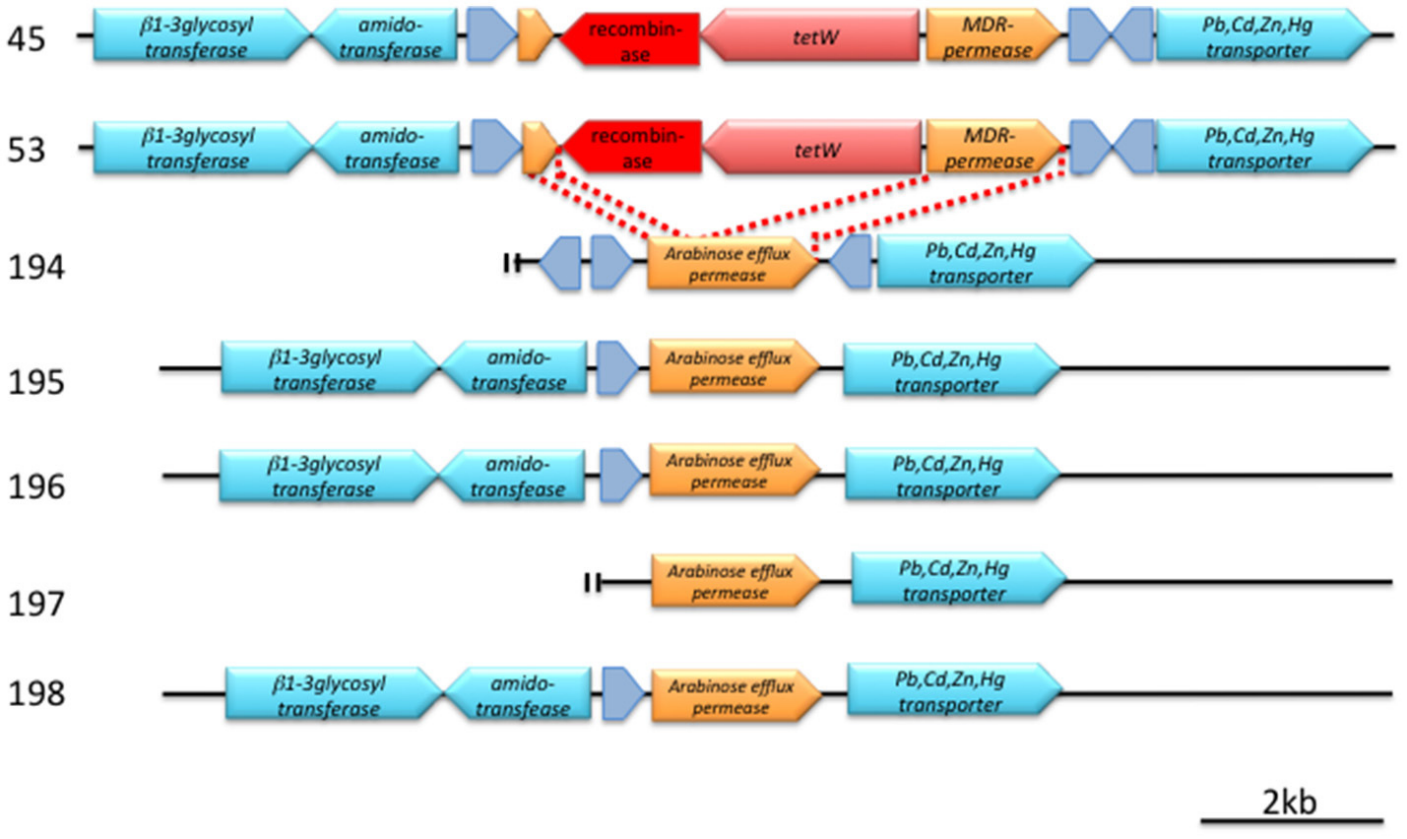
Detailed structure of a conserved genetic region around *tetW* (pink arrow) and a putative recombinase (red arrow). Hypothetical proteins are indicated by gray arrows. Annotation was made according to SEED. Bar indicates 2 kb length. For details, see text and Figure S1.

The *tetW* genes found in strains 45 and 53 are identical in DNA sequence, and both are located next to the same gene that encodes a serine recombinase (Figure 2, Figure S1). The *attL/R*-like direct repeat sequences could not be found around this *tetW*–serine recombinase gene cassette. The GC content of this cassette was 53.8%, which is lower than those in the flanking 4,000-bps region (59.4%). A BLASTN search for this serine recombinase coding gene (1,203 bases) indicated high similarities (identity = 100%, coverage = 100%, E-value = 0.0) with the transposase of *Treponema succinifaciens* DSM2489 (CP002631) and, to a slightly lesser extent (identity = 100%, coverage = 96%, E-value = 0.0), with that of *Lactobacillus amylovorus* 30SC (CP002559).

A comparison of the *tetW* cassette–flanking regions of strains 45 and 53 with the analogous regions of *tetW*-negative strains revealed that the cassette has been inserted in a gene encoding an arabinose efflux permease family protein, and the 3′-region of this permease protein gene was predicted to be an MDR permease by SEED (Figure 2, Figure S1).

## Discussion

We isolated seven strains of *Bifidobacterium* from pigs and wild boars. These isolates were identified as *B. thermacidophilum* according to their 16S rRNA sequences. *Bifidobacterium* is not a primary lactic acid bacteria in the pigs (Mitsuoka and Kaneuchi, 1977; Ushida et al., 2016). In the literature, *B. globosum, B. pseudolongum, B. thermophilum, B. boum*, and *B. choerinum* were detected from pig feces, and *B. boum* was a predominant *Bifidobacterium* in piglets (Mikkelsen et al., 2003). However, these species have a wide host range and are not limited to pigs (Scardovi et al., 1979; Yaeshima et al., 1992; Jans et al., 2013). *B. thermacidophilum* has also been detected in pigs (Mølbak et al., 2007). Marti et al. (2009) demonstrated that *B. thermacidophilum* is a pig-specific *Bifidobacterium* and can be used as a specific marker for pig-manure contamination. Accordingly, *B. thermacidophilum* was regarded as a *Sus*-specific *Bifidobacterium* species in this study, although the type strain of *B. thermacidophilum* subsp. *thermacidophilum* was isolated from waste water from a soybean curd factory in Beijing (Dong et al., 2000).

According to the 16S rRNA phylogenetic tree, pig isolates 45 and 53 were close to RBL67. These three strains and another pig isolate, 194, were close to both *B. thermacidophilum* subsp. *porcinum* and *B. thermophilum* JCM 1207^T^.

Since *B. thermacidophilum* is a recognized species of the very close relative of *B. thermophilum* (Dong et al., 2000), their close relationship is not surprising.

However, at least the phylogenetic position of *B. thermophilum* RBL67, a human infant isolate, is confusing and should be reclassified. In fact, the low ANI value (88%) shown by the RBL67 genome with that of *B. thermophilum* JCM 1207^T^ clearly indicates the distant phylogenetic relationship between them. Kheadr et al. (2007) listed this particular strain under the D group of *B. thermacidophilum* subsp. *porcinum*. We constructed phylogenetic trees based on draft genome sequences in this study: a maximum-likelihood phylogenetic tree of the amino acid sequence of the single-copy core CDSs (Figure 1B), a maximum-parsimony tree based on all of the SNPs (Figure 1C), and a pan-genome dendrogram (Figure 1D). All of these trees showed the same topology and several inconsistencies with a tree constructed by 16S rRNA sequences due to the insufficient resolution of 16S rRNA sequence alignment at subspecies level as indicated by Lugli et al. (2014). In draft genome-based phylogenetic trees, pig isolate 194 and *B. thermophilum* RBL67 were always placed in close proximity, and their relatedness to *B. thermacidophilum* subsp. *porcinum* JCM 16945 was suggested. Pig isolates 45 and 53 were always close to *B. thermacidophilum* subsp. *thermacidophilum* JCM 11165 rather than to *B. thermacidophilum* subsp. *porcinum* JCM 16945. Four wild boar isolates, 195, 196, 197, and 198, were always in the independent clade in these trees. The ANI values of around 93%, which was smaller than the 95% criteria for species identification, were shown by strains 195, 196, 197, and 198 with both *B. thermacidophilum* subsp. *porcinum* JCM 16945 and *B. thermacidophilum* subsp. *thermacidophilum* JCM 11165. This may suggest that these wild boar isolates could be reclassified in the future at the species level. In this study, we constructed a kSNP tree in addition to a core CDSs based analysis and a pan-genomic presence/absence matrix of the core genome to confirm the phylogenetic relationship of seven strains. In fact, the kSNP tree accurately represented the phylogenetic relationship of 58 *Streptococcus suis* strains in a previous study (Okura et al., 2017). Since the kSNP tree is based on k-mer selected as the optimal value predicted by the kSNP-associated Kchooser script, it does not consider the effect of recombination. The involvement of multicopy genes in the phylogenetic analyses may suggest different phylogenetic relationships according to which multicopy genes are selected. Therefore, we selected the single-copy genes for the phylogenetic analyses. Pseudogenes were also removed from the present analyses because individual pseudogenes had been under a different level of selection pressure. The concordant feature of pan-genomic analyses with the kSNP tree can represent the robust phylogenetic relationship of seven strains. Therefore, concordant results shown by phylogenetic trees (Figures 1B,C) and a dendrogram (Figure 1D) are not weak evidence for the phylogenetic relationship of these seven strains.

The separation of pig isolates from wild boar isolates depends on the biased distribution of genes (Tables S2, S3). This biased distribution may represent the adaptation of *B. thermacidophilum* to the environmental condition of the host intestine.

Obviously, the present genomic analyses were based on draft sequences, in which we can discuss the genes actually present, not absent, in the draft genome sequences. It is possible that many of the essential genes presumably present in the genome were missing. Therefore, we still need to complete the genome sequencing to confirm the present findings and following discussion. In addition, a recent study indicated that a small number of COGs for the carbohydrate metabolism represented in the core genome of *Bifidobacterium*, although the carbohydrate metabolism is the major functional family of COGs (Milani et al., 2014). This suggests the importance of analyses on paralogous genes for complete understanding of the adaptation of *Bifidobacterium* to the host environment. Although such limitations, several particular differences in gene distributions between two groups of isolates (the domestic pig cluster and the wild boar cluster) may indicate the relatedness of gene distribution in the genome to the feeding condition of the host. For example, the biased presence of fiber-degrading enzymes, endoglucanase, chitinase, pullulanase, and beta-mannosidase, is interesting because wild boars are omnivores whose diet is predominantly plant materials (Ballari and Barrios-Garcia, 2014). Cellulose and xylan comprise the major part of plant carbohydrates of the above-ground part, which comprises 70% of the plant materials consumed by wild boars (Ballari and Barrios-Garcia, 2014). Adaptation of these wild boar isolates to a fibrous diet was, therefore, suggested in comparison with isolates from domestic pigs that rely more on cereal starch than plant fiber. In addition to fiber-degrading enzymes, the possible presence of chitinase may reflect the dietary habit of wild boars, because fungi are the one of their major food items (Fournier-Chambrillon et al., 1995; Hohmann and Huckschlag, 2005). The unique presence of chitinase in wild boar isolates again suggests adaptation of the host food habit. Glucosidases and genes for a nucleic acid-base metabolism, such as cytosine deaminase, xanthine, and other nucleic acid-base permeases, were found to be genes associated with wild boar isolates, which suggests that a nitrogen-scavenging capacity is essential for the survival of intestinal bacteria under a protein-limited intestinal environment. This may reflect the protein-limited feeding condition of wild boars normally observed in the winter season (Genov, 1981). These particular profiles in the carbohydrate metabolism of wild boar isolates were shared by the type strain of *B. thermacidophilum* subsp. *thermacidophilum* and not by the pig-derived type strain *B. thermacidophilum* subsp. *porcinum*, which supports the adaptation of this species to the dietary habit of the host. Genes for iron metabolism were also distributed to wild boar isolates. These genes were not shared by pig isolates and the two type species of *B. thermacidophilum*. Since some of these genes were detected in a pig-derived *B. thermophilum* JCM1207 and human infant–derived *B. thermophilum* RBL67, it is inconclusive whether these iron metabolisms were related to the wild boar feeding habit.

The abundance of Type VI pilin (T4P) is also distinctive for wild boar isolates. T4P is generally displayed on the surface of Gram-negative bacteria such as *Pseudomonas aeruginosa* (Bradley, 1972) but also has been identified in several Gram-positive bacteria (Melville and Craig, 2013). DNA uptake was one of the functions of T4P system (Claverys et al., 2006). This also suggests the adaptation of wild boar isolates to the nitrogen-limiting condition that accelerates the nucleic acid metabolism in the GIT.

The mobile genetic elements associated genes were found more abundant in the pig isolates than in the wild boar isolates. This tendency was also found in the pig-derived type strain *B. thermacidophilum* subsp. *porcinum* (20 mobile element proteins) as compared to *B. thermacidophilum* subsp. *thermacidophilum* (10 mobile element proteins). Genes for the site-specific recombinase, integrase, transposase, and TrsK-like protein were detected in pig isolates, which suggests the capacity of a horizontal gene transfer. As a a barrier to horizontal transfer and acquisition of mobile genetic elements, Type 1 restriction enzymes (EC 3. 1. 21. 3. and EC 2. 1. 1. 72.) were deployed in pig isolates, but these genes were not shared by the type strains.

The CARD and SEED suggested the variety of drug-resistance genes. The genes for virulence and defense, such as the tetracycline resistance gene, *tetW*, and mercuric ion reductase (EC 1. 16. 1. 1.), and the lincomycin resistance gene were found to be pig-associated genes (under the intensive feeding system). A tRNA synthetase gene, *ileS*, can be considered to be involved in the natural resistance of *B. thermacidophilum* (Serafini et al., 2011). The similar tRNA synthetase gene, *alaS*, which is involved in novobiocin resistance, was suggested for all isolated strains, although *B. animalis* subsp. *lactis* and *B. longum* were susceptible to this antibiotic (D'Aimmo et al., 2007).

Disk diffusion tests for presumably acquired resistance showed a low level of concordance with the CARD and SEED predictions. Several fluoroquinolone resistance genes were suggested for all strains, including three type strains, but any resistance to ofloxacin was not proven. Although the macrolide resistance gene, *desR*, was suggested for wild boar isolates, they are all susceptible to erythromycin. Colistin resistance was also conferred for the strains harboring *PmrE*, but colistin resistance apparently was not related to the presence of *PmrE*. Fosfomycin resistance was conferred by the presence of *murA*, but the fosfomycin resistance was not concordant to the presence of *murA*. Such low level of concordance may be related to the lower level of similarities (≤ 60%), which suggests the detection of non-functional genes. In case of *gyrA* gene, SNPs were also suggested by CARD, which indicated the mutation within the gene. The concordance between the prediction and the disk susceptibility test was the tetracycline resistance. Therefore, we focused on tetracycline resistance in this study, although the tetracycline resistance in *Bifidobacterium* is well-known (Moubareck et al., 2005). The biased distribution of *tetW* to strains 45 and 53, from a farm where tetracycline is heavily used, is noteworthy, because the isolates from a pig under a drug-free feeding condition and from wild boars were free of *tetW* and susceptible to tetracycline. While *Bifidobacterium* possesses a naturally occurring resistance to aminoglycoside antibiotics such as gentamycin (Moubareck et al., 2005; Ammor et al., 2008), acquired resistance was suggested for tetracycline and macrolide among the known antimicrobials (Moubareck et al., 2005; Van Hoek et al., 2008, 2011). Among the presently studied seven isolated strains, only two strains, which were isolated from pigs in a commercial pig farm with routine tetracycline use, were found to possess the *tetW* gene. In the literature, strains of *B. adolescentis, B. animalis lactis*, and *B. pseudocatenulatum*, mostly human-associated species, showed relatively high resistance (≥32 mg/L) to tetracycline due to the acquisition of *tetW* (Masco et al., 2006; Ammor et al., 2008). *B. thermophilum* strains of a pig or cattle origin also showed very high resistance to aminoglycoside but with varying degrees of resistance to tetracycline (Mayrhofer et al., 2011).

The *tetW* gene is now widely distributed to the genus *Bifidobacterium* in humans and livestock (Masco et al., 2006; Aires et al., 2007). The *tetW* gene product detected in the present study showed high similarity (>99%) to many *tetW* sequences (>50 sequences) deposited in the public database (BLAST search was executed on March 1, 2016, through the DDBJ website). Because of the high sequence similarly between the *tetW* genes identified so far, it has been suggested that *tetW* has recently appeared and rapidly spread among various bacteria (Scott et al., 2000). In fact, the *tetW* genes previously detected in *B. longum* and *Bifidobacterium* sp. ISO3519 are most closely related to the *tetW* found in other bacterial species, such as *Megasphaera elsdenii, Roseburia hominis, Mitsuokella multocida*, and *Butyrivibrio fibrisolvens*, inhabiting animals and humans (Aminov and Mackie, 2007).

A serine recombinase is often associated with integrative genetic elements (Bibb et al., 2005). Although *attL/R*-like direct repeat sequences could not be found around this *tetW*–serine recombinase gene cassette, this cassette may represent a mobile genetic element that mediates the horizontal transfer of *tetW*. The GC content of this cassette was 53.8%, which is lower than those in the flanking 4,000 bps region (59.4%). Such a difference in GC content also suggests the horizontal transfer of *tetW*.

The presence of this serine recombinase in *Bifidobacterium* was not known before, but this recombinase showed high similarity with those of *T. succinifaciens* DSM2489 and *L. amylovorus* 30SC, both of which were isolated from pigs (Cwyk and Canale-Parola, 1979; Oh et al., 2011). Although *T. succinifaciens* DSM 2489 also contains the *tetW* gene, it is 20 kb apart from the serine recombinase gene. Therefore, the relationship of these two genes in *T. succinifaciens* DSM 2489 is unknown.

A comparison of the *tetW* cassette–flanking regions of strains 45 and 53 with the analogous regions of *tetW*-negative strains revealed that the cassette has been inserted in a gene encoding an arabinose efflux permease family protein, and the 3′-region of this permease protein gene was predicted to be an MDR-permease by SEED (Figure 2, Figure S1).

The presently detected two *tetW* genes were identical. Moreover, the serine recombinase was also identical for both pig isolates, with a minor difference in an intergenic spacer sequence. The presence of a common origin for the *tetW* gene cassette in these two isolates was suggested. Since strains 45 and 53 are phylogenetically very close, it is, therefore, plausible that these two strains are progeny of a strain of *B. thermacidophilum* that horizontally received this *tetW* cassette from an unknown origin sometime in the past.

*Bifidobacterium* is the potential target of probiotics (Sanders, 2008; Amagase, 2011), since the transferable drug resistance associated with this bacterium is a serious concern for the safety of their probiotic use (Gueimonde et al., 2013). Tetracycline is still frequently used in animal agriculture, particularly in pig production in many countries, including Japan (Itoh, 2014; Van Rennings et al., 2015). Therefore, there is still a considerable risk of tetracycline resistance dissemination into human society through the food supply (Barbosa et al., 1999; Patterson et al., 2007; Kazimierczak et al., 2009). In this circumstance, *B. thermophilum* that is detected in both pigs and humans may become a more direct threat to our society than the presently analyzed *B. thermacidophilum*. In fact, *B. thermophilum* JCM 1207^T^ was proven to harbor a functional *tetW*. However, *B. thermophilum* RBL67, which was isolated from a human infant, was actually very close to our pig isolates, *B. thermacidophilum* strains 45 and 53, according to phylogenetic analyses on a draft genome basis. This suggests that a certain *B. thermacidophilum* can become a member of the human intestinal microbiota. The possible risk of tetracycline resistance dissemination into human society by pig-origin *B. thermacidophilum* cannot be ruled out.

## Conclusion

One of the important differences between pig-derived isolates (under the intensive feeding system) and wild boar–derived isolates was that the former possessed a horizontally transferred *tetW* gene cassette with a putative serine recombinase, though the mobility of this *tetW* cassette is yet to be determined. Although *B. thermacidophilum* can be recognized as a pig-specific *Bifidobacterium* species, *B. thermacidophilum* subsp. *porcinum* and its close relative, *B. thermophilum*, have been recognized in both humans and pigs (Kheadr et al., 2007; Mayrhofer et al., 2007; Von Ah et al., 2007). This suggests a potential risk for the transmission of tetracycline resistance to human intestinal *Bifidobacterium* through the food chain. It is clear that the routine use of tetracycline in pig farms supports the continuous presence (or selection) of *tetW*-positive *B. thermacidophilum*, because a strain from a sow in our experimental farm, where no antimicrobials were fed, was free from *tetW* resistance gene, together with those derived from wild boars, which were likely to feed on antimicrobial-free natural diets.

## Author contributions

ST contributed to the design of the study, isolation of strains, and physiological analyses on bacteria and interpreted the data and wrote the initial draft of the manuscript. FM contributed to the Bioinformatic analysis and interpretation of data, and assisted in the preparation of the manuscript. YO and AT contributed to the sequencing by Next-generation sequencer and interpretation of the data. TH and MO contributed the critical revision of the articles for important intellectual content respectively for genome analyses and for phylogenetic analyses. KU contributed to the Conception and design of the study, physiological analyses, and data interpretation. The final version of the manuscript was approved by all authors.

### Conflict of interest statement

The authors declare that the research was conducted in the absence of any commercial or financial relationships that could be construed as a potential conflict of interest. The reviewer ABF and handling Editor declared their shared affiliation, and the handling Editor states that the process nevertheless met the standards of a fair and objective review.

## Supplementary material

Figure S1 Sequence comparison between *Bifidobacterium thermacidophilum* pig isolates and wild boar isolates. Genomic regions around *tetW* (tet) and its mobile element (tr) were compared to reveal the insertion in a parallel view. Figures were drawn by GenomeMatcher (Ohtsubo et al., 2008).

Table S1 Average Nucleotide Identities of Bifidobacterim isolates from pigs and wild boars with type strains. Caliculation was made at http://enve-omics.ce.gatech.edu/ani/ (Goris et al., 2007).

Table S2 SEED category features for genomes of seven *Bifidobacterium thermacidophilum* strains (number of genes in draft genomes).

Table S3 Genes with host animal-biased appearance frequencies in genomes of seven strains of *Bifidobacterium thermacidophilum* (number of gene copy in draft genomes) and laboratory strains.
