# Supplementary figures and images for: Genomic Characteristics of Bifidobacterium thermacidophilum Pig Isolates and Wild Boar Isolates Reveal the Unique Presence of a Putative Mobile Genetic Element with tetW for Pig Farm Isolates
Source: Front Microbiol. 2017 Aug 15;8:1540. doi: 10.3389/fmicb.2017.01540 (PMC5561799; doi:10.3389/fmicb.2017.01540)

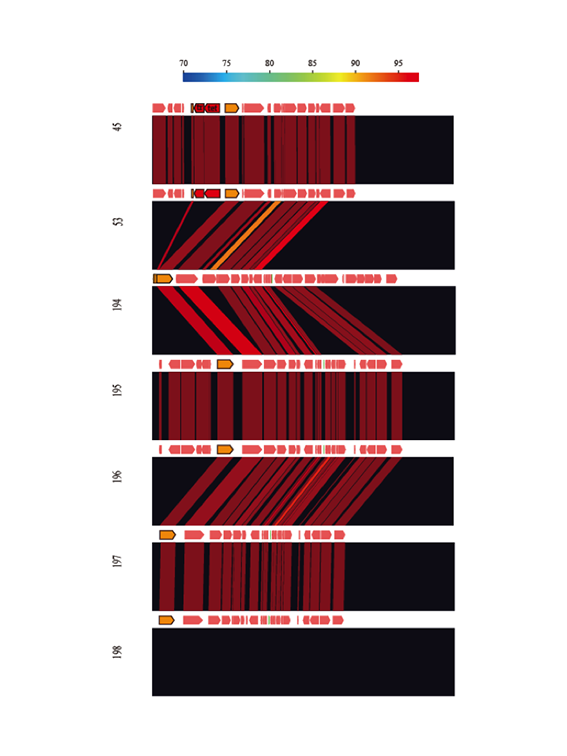

Supplement: Figure S1 — Sequence comparison between Bifidobacterium thermacidophilum pig isolates and wild boar isolates. Genomic regions around tetW (tet) and its mobile element (tr) were compared to reveal the insertion in a parallel view. Figures were drawn by GenomeMatcher (Ohtsubo et al., 2008). [file Image1.TIFF]
